# Micro and nano hierachical structures of BiOI/activated carbon for efficient visible-light-photocatalytic reactions

**DOI:** 10.1038/s41598-017-12266-x

**Published:** 2017-09-15

**Authors:** Jianhua Hou, Kun Jiang, Ming Shen, Rui Wei, Xiaoge Wu, Faryal Idrees, Chuanbao Cao

**Affiliations:** 1 Jiangsu Key Laboratory of Environmental Material and Engineering, College of Environmental Science and Engineering, Yangzhou University, Yangzhou, 225127 P. R. China; 2 College of Chemistry and Chemical Engineering, Yangzhou University, Yangzhou, 225002 P. R. China; 3 Research Centre of Materials Science, Beijing Institute of Technology, Beijing, 100081 P. R. China

## Abstract

Constructing the heterojunctions or designing the novel nanostructures are thought as effective methods to improve photocatalytic activities of semiconductors. Herein, a one-step green route was developed to fabricate bismuth oxyiodide/activated carbon (BiOI/C) composite. The prepared BiOI/C exhibit obviously red shifts and increased absorption range of visible light. The presence of Bi-C bonds confirms the heterojunction, on account of which the BiOI nanosheets tightly grew on the surface of carbon and subsequently provided the hierarchical structure, sufficient interfacial interaction and high specific surface area. Significantly, the sufficient interracial interaction is beneficial to the detachment of electrons (e^−^)-holes (h^+^) pairs and the Bi-C bonds work like a bridge to rapidly transmit the e^−^ from BiOI to carbon. What’s more, the hierarchical structure of BiOI/C efficiently shortened the diffusion pathways of pollutants and the high S_BET_ provided more exposed reaction sites. Benefiting from multiple synergistic effects, the as-prepared BiOI/C exhibited enhanced photocatalytic activities in degrading Rhodamine B (RhB) solution under visible light irradiation. The degradation rate of optimized BiOI/C reaches up to 95% in 120 min, and the efficiency is 3.36 times higher than pure BiOI. This study provides a promising strategy that activated carbon can be utilized in highly-efficiency photocatalysts.

## Introduction

In past few years, the water pollution and energy crisis problems have seriously threatened the sustainable development of human beings^1^. Semiconductor photocatalysts have caught considerable attention because of their potential in solving energy and environmental problems^2–5^. Traditional semiconductor photocatalysts such as TiO_2_ and TiO_2_-based photocatalysts are extensively used to degrade these organic dyes in water^6^. However, their relatively large band gaps greatly hindered the absorption range of visible light^7^, which involved the high cost and complex degradation process. It is the demand of fast growing society to exploit new semiconductor photocatalysts with suitable band gap, easy synthesis techniques and favorable photocatalytic properties^8,9^. Among newly found semiconductor photocatalysts, bismuth oxyhalide (BiOX, X = Cl, Br, I) has drawn people’s attention due to their layered tetragonal crystal structure and suitable band gaps^10–14^, which are favorable optical properties for degradation pollutants under extensively available sunlight.

Among BiOX, BiOBr and BiOCl mainly respond to UV light, while BiOI shows a high utilization rate of visible light due to narrow band gap (1.72–1.9 eV)^15,16^. Nevertheless, the low quantum yield, weak photo-oxidation ability and easy recombination of e^−^–h^+^ pairs of BiOI still hinder its photocatalytic property^17^. Regarding these drawbacks, different strategies have been adopted to improve photocatalytic response of BiOI, such as, (i) designing a novel nanostructures to increase S_BET_ to provide more active sites for reaction^18,19^; (ii) constructing a hierarchical structures to shorten the pathways of water pollutants^20,21^; (iii) establishing a heterojunction with other semiconductors to prevent the recombination of the e^−^–h^+^ pairs and to facilitate the advance quantum efficiency^22,23^. It’s no doubt that the aforementioned strategies provide a great improvement in photocatalytic response of BiOI.

Combining BiOI with fabricated porous carbon material is possible to meet the features required for improving the photocatalytic activity. BiOI/carbon composites *e.g*. BiOI/graphene^24,25^, BiOI/carbon nanotubes^26^ and BiOI/g-C_3_N_4_^27,28^ exhibited enhanced photocatalytic response. It is suggested that functional porous carbon material can improve the photocatalytic activity due to their good electron transfer ability, favorable chemical stability and high S_BET_. However, most of the reported BiOI/carbon composites still suffer some drawbacks, *i.e*. the graphene and carbon nanotubes for their activation undergoes to the carboxylation or acid purification pretreatments^29^. These pretreatments are complicated, toxic and high costly, which might limit the applications of BiOI/carbon photocatalysts. On the contrast, the commercial activated carbon with high S_BET_^30^ can effectively overcome this problem. By compositing it with BiOI *via* a green, facile and low cost method, the BiOI/C is expected to show broadened absorption range of visible light, as well as enhanced photocatalytic property. In addition, the absorption activity of activated carbon will improve the removal of organic contamination.

**Figure 1:**
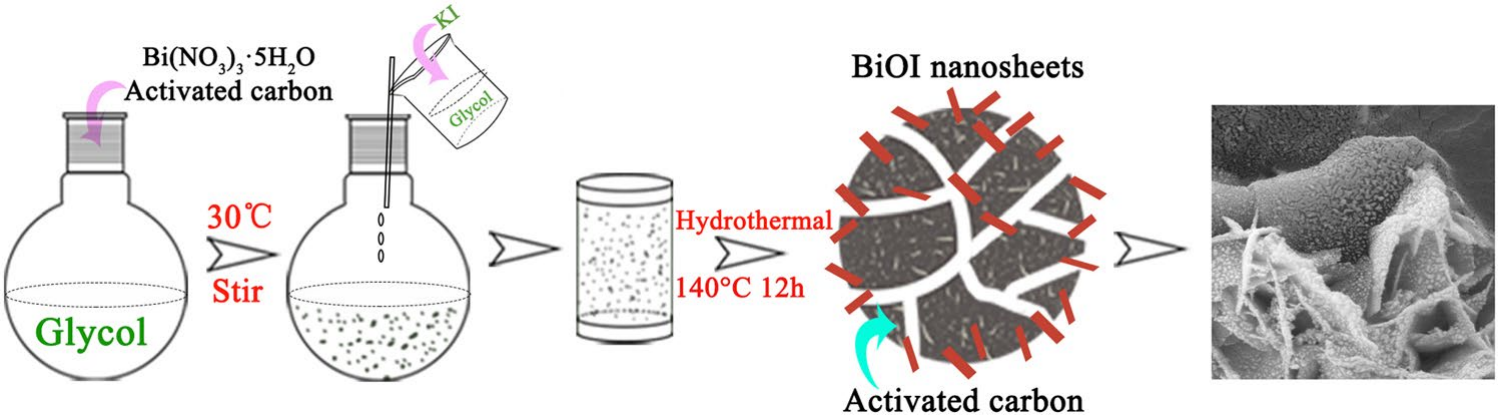
Schematic illustration of the preparation of BiOI/C composites micro-nano-hierarchical structures.

In this work, a commercial activated carbon (YP-17D) with high S_BET_ (1660 m^2^/g)^31^ was used to synthesize BiOI/C composites micro-nano-hierarchical structures by a one-step green method. The general preparation process of BiOI/C is illustrated in Fig. 1. The as-obtained a BiOI/C possess favorable features including heterojunction, hierarchical organization, sufficient interfacial interaction sites and high specific surface area (S_BET_ = 86.8~145.6 m^2^/g), which endow the BiOI/C with easy separation of e^−^h^+^ pairs, high quantum efficiency, short diffusion pathways of pollutants and adequate reaction sites. The multiple synergistic effects of these characteristics dramatically improve the photocatalytic property of BiOI/C for water pollutants degradation.

## Result and Discussion

### The Crystal Structure and Composition

**Figure 2:**
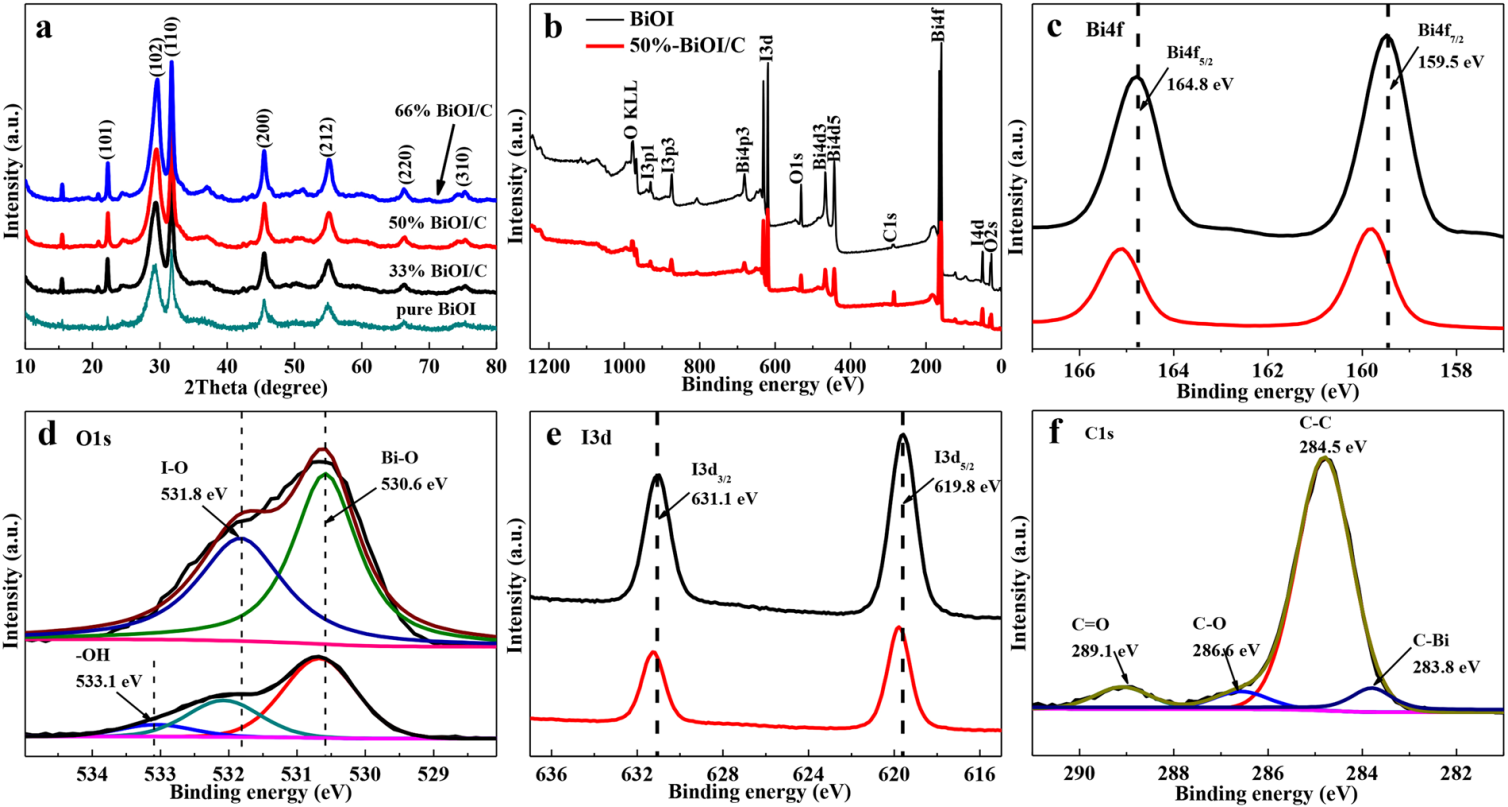
(**a**) XRD patterns of BiOI and X-BiOI/C. (**b**–**e**) XPS spectra of BiOI and 50%-BiOI/C for full survey, Bi 4 f, O 1 s and I 3d. (**f**) XPS spectra of 50%-BiOI/C for C 1 s.

The XRD spectrum of BiOI and BiOI/C is shown in Fig. 2a, the diffraction peaks of BiOI at 24.3, 29.6, 31.6, 45,4, 55.1, 66.1 and 75.1° are corresponding to (101), (102), (110), (200), (212), (220) and (210) plane, respectively, which well match with the tetragonal phase structure (JCPDS No.10-0445)^28^, demonstrating the high purity of the sample. The XRD patterns of activated carbon (Figure S1) exhibit two broad peaks at 22° for (002) and 43° for (101) plane, which correspond to the previous studies of the amorphous carbon^32^. Meanwhile, no characteristic peaks assigned to activated carbon are observed, which might be due to the relatively low amount and weak diffraction intensity of activated carbon in the BiOI/C^12^. However, the half widths of BiOI/C express slightly increase contributing to the decreased crystallinity, and the half width of (102) and (110) peaks is 1.966 and 0.529, respectively. Importantly, the exposed (110) facets were reported to be favorable for adsorbing O_2_ and forming more O_2_^**·−**^ radicals^33^, thus effectively improving the photocatalytic properties of BiOI/C. The experience of O_2_^**·−**^ was proved by the following active species trapping experience. The XPS spectra of BiOI and 50%-BiOI/C were carried out to evaluate the valence state of surface elements. The full survey spectrum (Fig. 2b) of BiOI indicates the presence of Bi, O and I atoms, a small peak at about 285 eV is also present in the sample due to carbon pollutant. For 50%-BiOI/C, besides Bi, O and I, a strong diffraction peak of C can be observed due to the addition of YP-17D. The Bi 4 f spectrum of BiOI (Fig. 2c) exhibits two diffraction peaks at 159.5 and 164.8 eV, which can be assigned to Bi 4 f_7/2_ and Bi 4f_5/2_ of [Bi_2_O_2_]^2+^, respectively^34,35^. Importantly, the Bi 4f peak of 50%-BiOI/C slightly shift (0.3 eV) as compared to BiOI indicating the surface chemical environment changing of Bi due to the interaction between carbon and BiOI^11,12^. The O1s spectra (Fig. 2d) of BiOI and 50%-BiOI/C at 530.6 eV and 531.8 eV are compliance with Bi-O bonds of [Bi_2_O_2_]^2+^ and I-O bonds^34,36^. Furthermore, the peak at 533.1 eV of 50%-BiOI/C agrees well with hydroxyl functional groups of carbon. About 0.1 eV shift in I 3d spectrum of 50%-BiOI/C (Fig. 2e), further revealing the interaction between carbon and BiOI. The C1s spectra (Fig. 2f) of 50%-BiOI/C can be divided into four different peaks. The binding energies at 284.5, 286.6 and 289.1 eV are corresponding to C-C, C-O and C=O, respectively. Interestingly, the small peak at lower binding energy of about 283.8 eV in C 1 s spectrum is usually referred to carburet in reported studies^11,37^, confirming the formation of Bi-C bonds. Further information of Bi-C bonds could be observed in following FTIR spectra. Deriving from bridge effects of Bi-C bonds, the BiOI nanosheets tightly grew on the surface of carbon to form sufficient interfacial interaction. The sufficient interfacial interaction can remarkably accelerate the separation of e^−^h^+^ pairs to generate free radicals (O_2_^·−^,^·^OH). Then these radicals degrade organics into micro-molecule such as H_2_O and CO_2_ through photocatalytic redox reactions, as a result, improving the photocatalytic properties.

**Figure 3:**
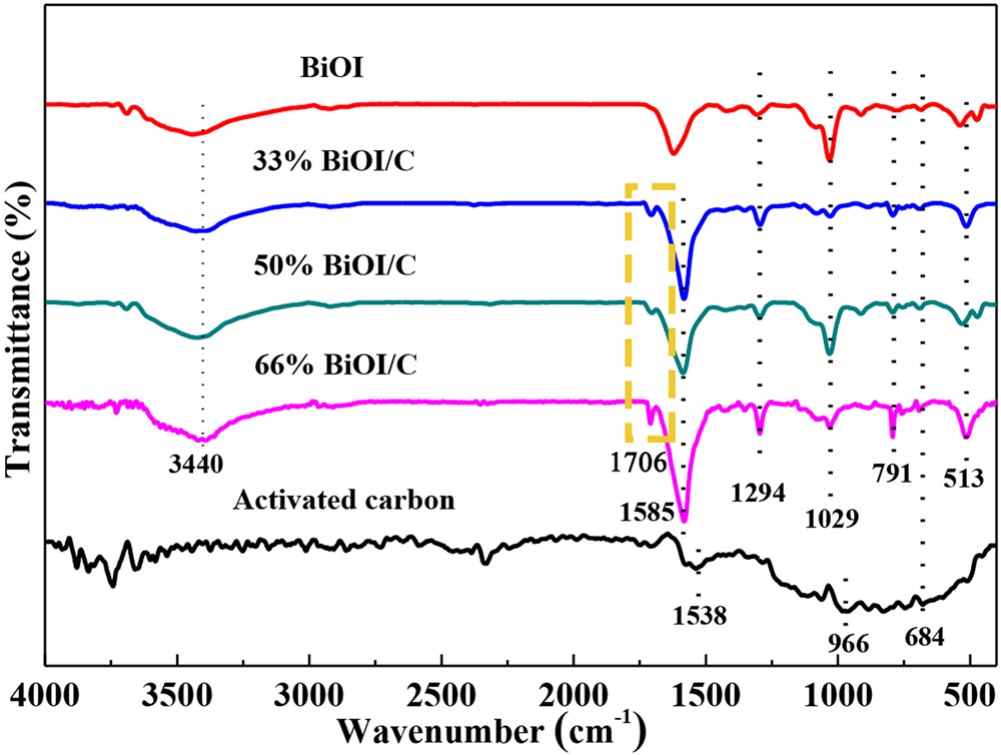
FT-IR analysis of YP-17D activated carbon and BiOI/C.

The FT-IR analysis was shown in Fig. 3 to further evaluate the surface functional groups of the as-prepared materials. For pure BiOI, the characteristic absorption bands below 600 cm^−1^ and around 3340 cm^−1^ attribute to the vibration mode of Bi=O=Bi and O-H stretching vibrations of absorbed water molecules^5^, which are also observed in BiOI/C samples. For carbon, the characteristic absorption bands locate at 684, 966, 1538 and 1706 cm^−1^ are correspond to C-O-C, C-OH, O-H and C=O stretching vibrations of the -COOH group, demonstrating the abundant oxygen-containing functional groups^11,12,24^, which derives from incomplete carbonization. After compositing carbon with BiOI, the characteristic peaks of BiOI/C express little differences with pure BiOI except a new peak appearing at 1706 cm^−1^, which indicates that the -COOH of carbon react with the surface hydroxyl of BiOI and successfully form chemically bonded BiOI/C composites and further prove the formation of Bi-C bonds^5,24^.

### Microstructure and Morphology Analysis

**Figure 4:**
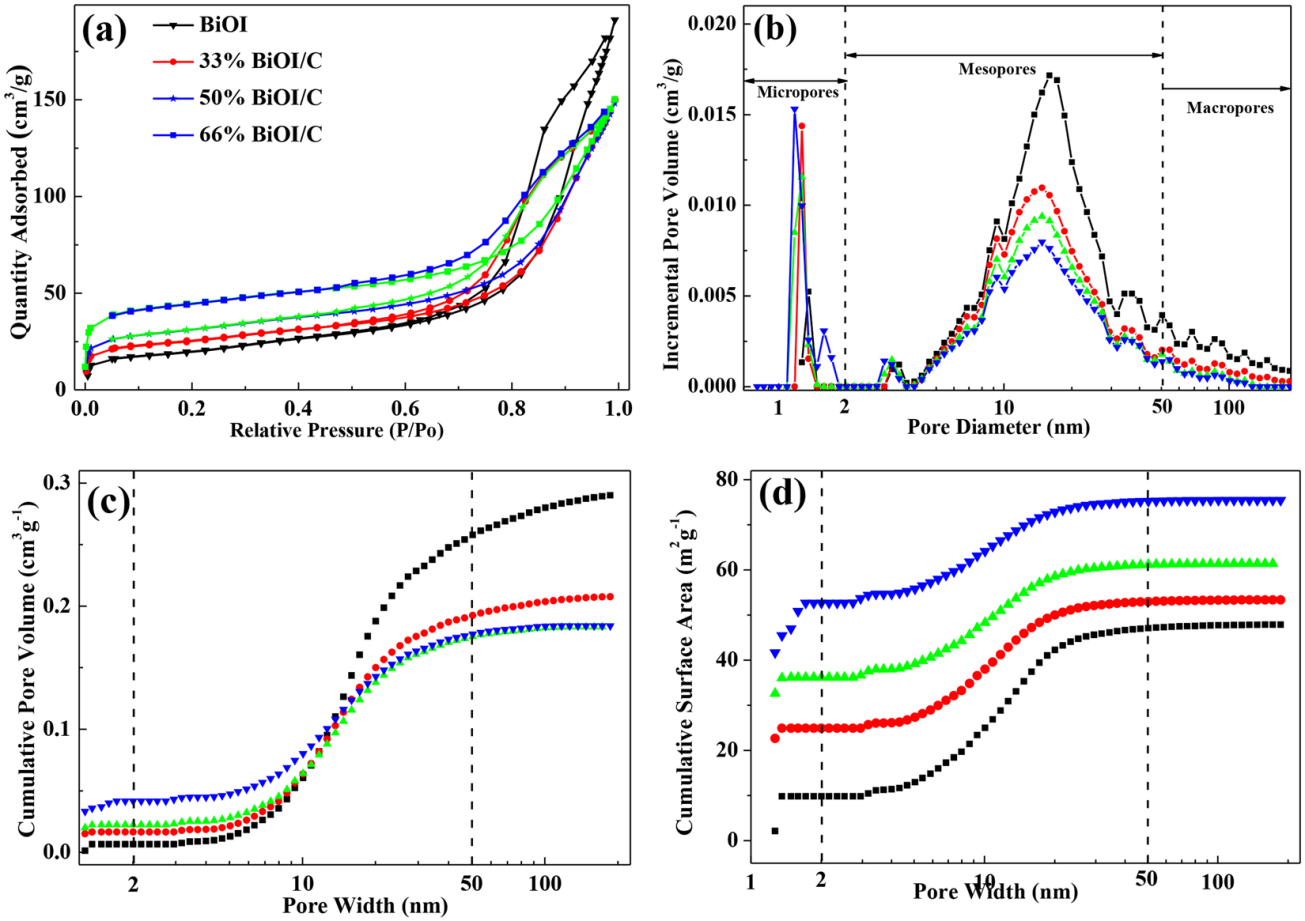
(**a**) Nitrogen adsorption-desorption isotherm (**b**) Pore size distribution (**c**) Cumulative pore volume and (**d**) Cumulative surface area of BiOI and BiOI/C.

The nitrogen adsorption-desorption isotherms of pure BiOI and BiOI/C possess hysteresis loops with a wide P/P_0_ range about 0.5–1.0, indicating the typical mesopores features (Fig. 4a). The pore size distribution was calculated by utilizing density Functional theory (DFT) (Fig. 4b). The micropores range in 1.1–1.7 nm and the optimized mesopores center of 18.3 nm, which clearly demonstrate the hierarchical pores structure of BiOI/C. Figure 4c,d shows the cumulative pore volume and cumulative pore area, respectively. The percentage of micropores, mesopores and macropores are displayed in Tables S1 and S2, which also demonstrate the hierarchical pores structure. Compared with YP-17D (Figure S2), the pore size less than 1.1 nm almost disappear in BiOI/C, and the total micropore area significantly decrease from 1262.6 m^2^/g to 34.1 m^2^/g. It indicates that BiOI have effectively grown on the surface of porous carbon due to Bi-C bonds and endow the BiOI/C with sufficient interfacial interaction, which is favorable for separating and transmitting e-h^+^ pairs^5,24^. The mesopores on the accumulated BiOI nanosheets are beneficial for the pollutants entering the pore tunnel^38^. Then, attributing to the confinement effect, the pollutants can be trapped within the effective functional mesoporous pores where photo-electrons accelerate the degradation process. The S_BET_ of 50%-BiOI/C reaches up to 145.6 m^2^/g, which is 2 time of pure BiOI (70.1 m^2^/g), and much higher than other studies^5,18,37,39–41^. The main reason for the increased S_BET_ is that the high viscosity of glycol slow the reaction of Bi(NO_3_)_3_·5H_2_O and KI, thus prompting the growth of BiOI in the form of nanosheets. The high S_BET_ can offer more reaction sites for degrading pollutants, thus improving photocatalytic property.

**Figure 5:**
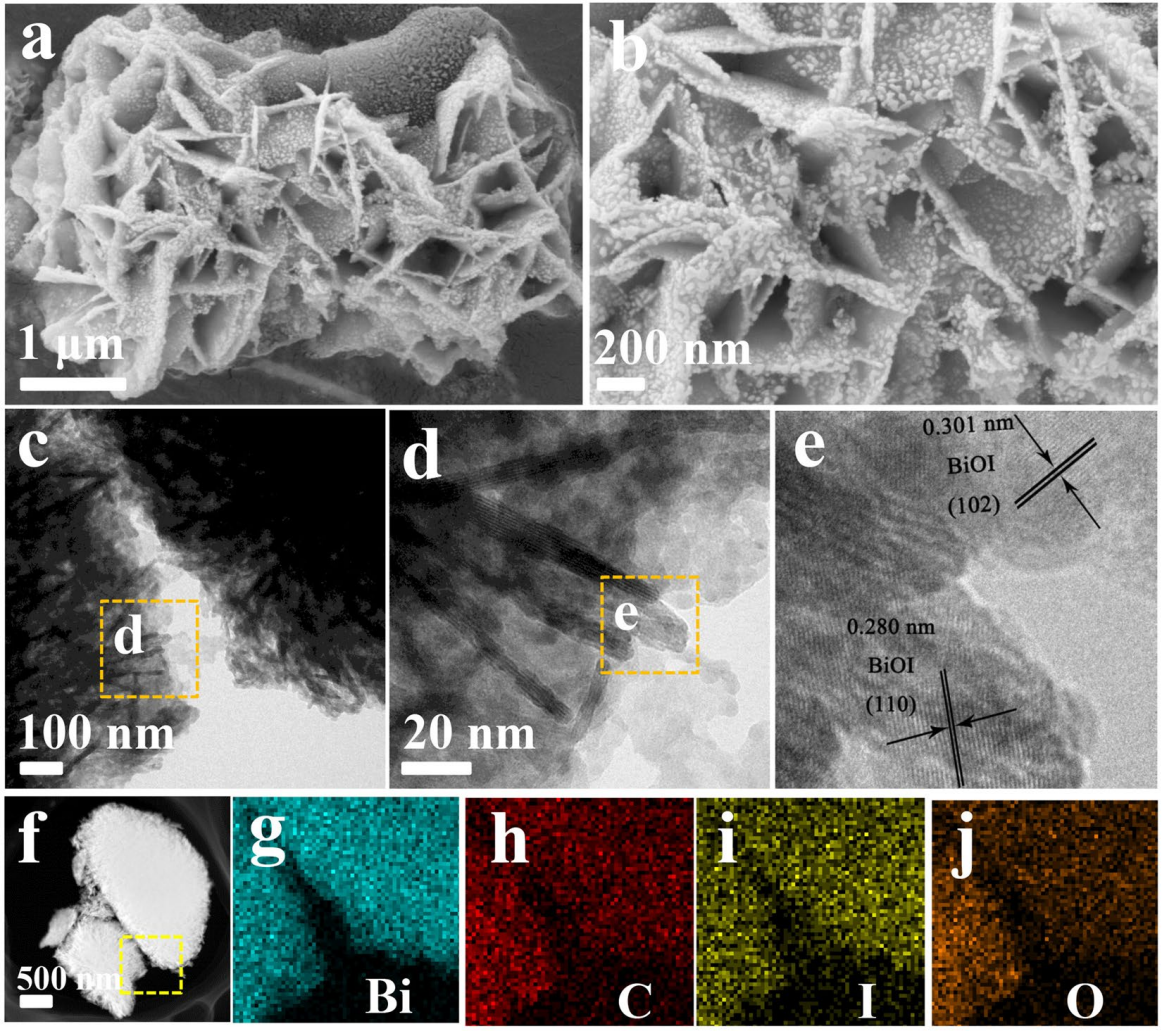
(**a**,**b**) SEM images, (**c**–**e**) HRTEM images and (**f**–**j**) the corresponding EDX elemental mapping of 50%-BiOI/C.

To further evaluate the morphology, the 50%-BiOI/C was taken for instance and characterized by SEM and HRTEM. It can be clearly observed from SEM images (Fig. 5a,b) that BiOI nanosheets have grown on the carbon surface to form the unique hierarchical structure, and BiOI particle further (diameter about 10 nm) deposit on the nanosheets. The thickness of BiOI nanosheets is about 10–30 nm. The lattice fringes with a spacing of 0.301 nm and 0.280 nm in HRTEM images (Fig. 5c–e) can be indexed to (102) and (110) planes, respectively, which is consistent with the XRD patterns. Interestingly, BiOI nanosheets work like numerous mirrors to reflection and absorption of incident light, thus efficiently increasing the production of photon-electrons. The hierarchical structure of BiOI/C can greatly shorten the diffusion pathways of pollutants, which is favorable for higher utilization efficiency of reaction sites. The elemental mapping analysis (Fig. 5f–j) demonstrates the uniform distribution of Bi, C, I and O elements. The SEM images of other BiOI/C are displayed in Figure S3 and the morphology is roughly the same with the 50%-BiOI/C.

### Optical Properties

**Figure 6:**
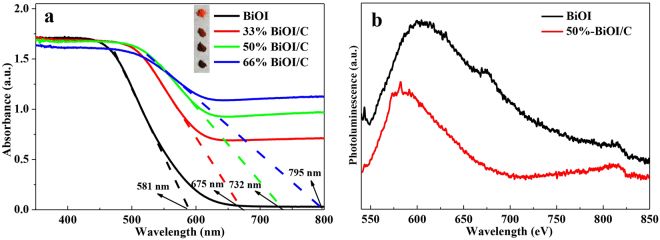
(**a**) UV-Vis diffuse reflectance spectra (inset: corresponding photographs) and (**b**) photoluminescence spectra of BiOI and 50%-BiOI/C.

The UV-Visible diffuse reflectance spectroscopy (UV-DRS) with a wavelength range of 200–800 nm is used to detect the optical properties of BiOI and BiOI/C as displayed in Fig. 6a. The inset photograph of BiOI, 33%-BiOI/C, 50%-BiOI/C and 66%-BiOI/C in Fig. 6a shows the gradually colors changes (dark) from brick-red to brown with the increasing contents of carbon. The darker of the sample is, the more light it will absorb. Theoretically speaking, carbon works as visible-light photosensitizer to BiOI in BiOI/C due to the efficient chemical bonds, and the increasing amount of carbon can improve the interaction between carbon and BiOI, finally enhancing the harvest of visible light^42,43^. Therefore, the BiOI/C composites exhibit obvious enhancement in absorbing visible light compared to pure BiOI and the absorption edges red shift from 581 to 675, 732 and 795 nm. Considering the dominant status of visible light in solar energy, it is suggested that BiOI/C may possess delightful photocatalytic activity. The photoluminescence spectrum (PL) of BiOI and 50%-BiOI/C were measured under an excitation of 532 nm to further investigation of the recombination of e^−^–h^+^ pairs. As shown in Fig. 6b, a broad emission peak of 50%-BiOI/C at approximately 570–650 nm derives from the recombination of e^−^–h^+^ through band transitions. The much lower peak intensity of 50%-BiOI/C indicates a higher separating capacity of e^−^–h^+^ pairs as compared to BiOI, suggesting that the addition of carbon efficiently enhance the interfacial charge transfer and hence favors the photocatalytic process^11,26,39^. The plots of (αhv)^1/2^ versus hv and corresponding band gaps were shown in Figure S4 for further investigating the VB/CB levels of as-prepared materials.

### Photocatalytic Activities and Mechanism

**Figure 7:**
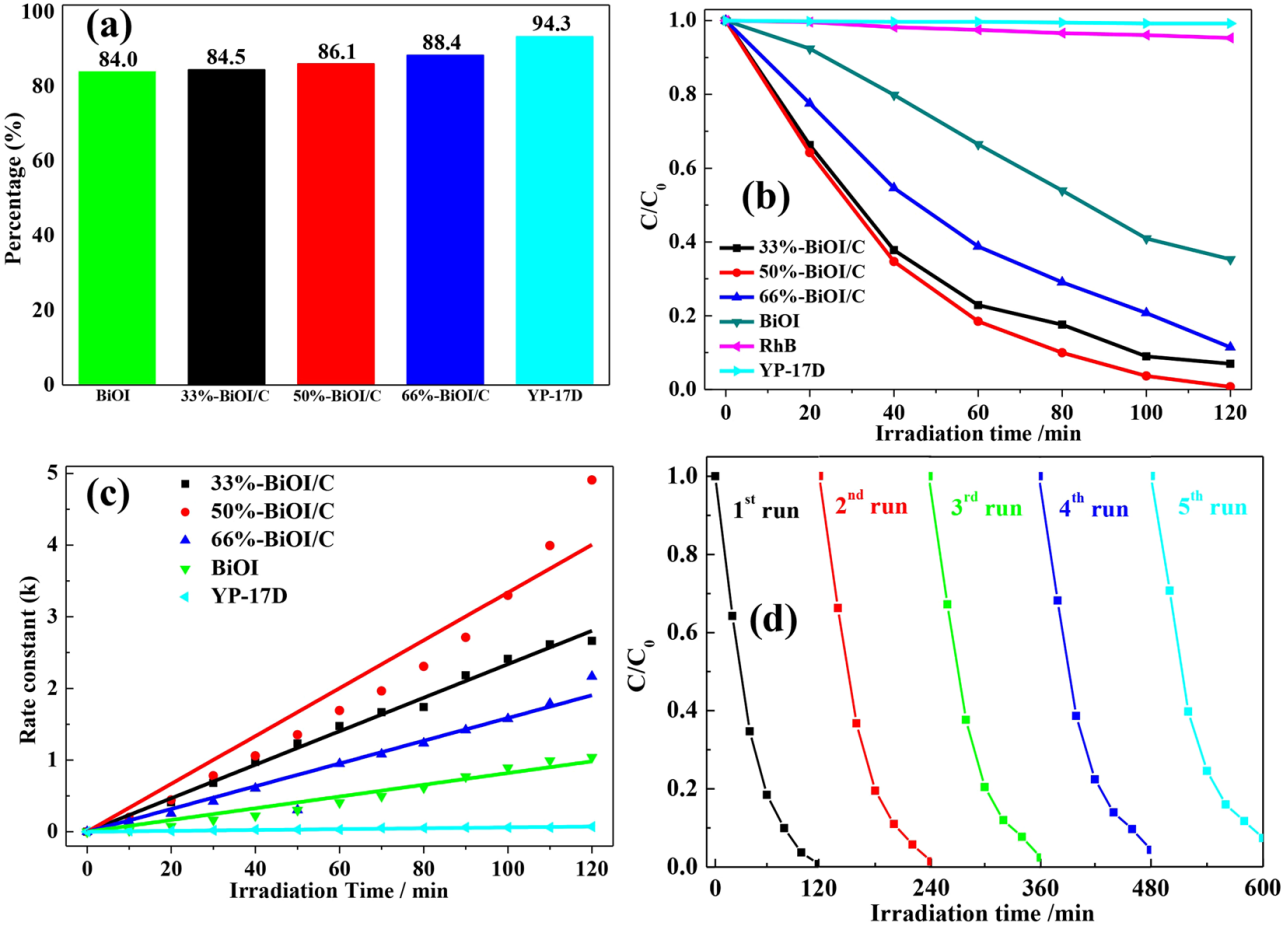
(**a**) Absorption activities. (**b**) Photocatalytic activities. (**c**) Kinetic study of YP-17D, pure BiOI and X-BiOI/C and (**d**) Long-term cyclic experiment of 50%-BiOI/C in degradation of RhB under visible-light irradiation.

To evaluate the photocatalytic activities of as-prepared pure BiOI and BiOI/C, they were first mixed with RhB and dark absorption for 60 min. From Fig. 7a we could draw the conclusion that most of the RhB had been absorbed due to the high S_BET_ of as-prepared materials, and the absorption ability improved with the increasing mole ratio of activated carbon. The degradation of RhB is carried out under visible-light (500 W Xe lamp, λ > 420 nm). The degradation rate is calculated by using the following formula: photodegradation rate = (C_0_ − C)/C_0_, in which C_0_ and C are the original and degraded concentrations of dyes, respectively. Obviously, the self-degradation of RhB and degradation over YP-17D could be ignored without BiOI according to Fig. 7b. The pure BiOI absorbed about 65% of the RhB in 60 min, and removed about 64% of RhB in 120 min, while the number of 50%-BiOI/C reaches to 95%, showing the best photocatalytic activity. Further by increasing the quantity of the activated carbon (66%-BiOI/C), the degradation rate is decreased. It is attributing to the shielding effect^44^ of activated carbon, which hinders the adsorption of incident light. Figure 7c shows the kinetic study for the degradation of RhB with pure BiOI and BiOI/C, and the corresponding pseudo-first-order rate constant (k) is calculated by the formula: ln(C_0_/C) = kt, in which k and t are constants. It can be observed that the degradation process follows the pseudo-first-order kinetics and the rate constants for pure BiOI, 33%-BiOI/C, 50%-BiOI/C, 66%-BiOI/C are 0.00868, 0.0181, 0.0235 and 0.0292 min^−1^. It’s obvious that 50%-BiOI/C shows the fastest rate constant, and the value is about 3.36 times greater than pure BiOI. The photocatalytic activities of BiOI/C are superior or comparable to many other BiOX-based photocatalysts, including BiOI micro-flowers (26% RhB degraded within 120 min)^45^, BiOI hollow microspheres (93% RhB degraded within 120 min)^18^, 3D hierarchical graphene-BiOI nanoarchitectures (57% RhB degraded within 120 min)^46^, Core/Shell BiOI-BiVO_4_ composites (95% RhB degraded within 180 min)^47^, CQDs/BiOI hollow microspheres structure (68% RhB degraded within 120 min)^48^. The corresponding UV-Vis spectra were displayed in Figure S5. Furthermore, we have also measured the long-term cyclic performance of 50%-BiOI/C to detect the stability of our material. The result shown in Fig. 7d demonstrates a slightly decrease (<5%) in degradation of RhB under visible-light irradiation after 5 cycles, indicating the highly stability of the material.

**Figure 8:**
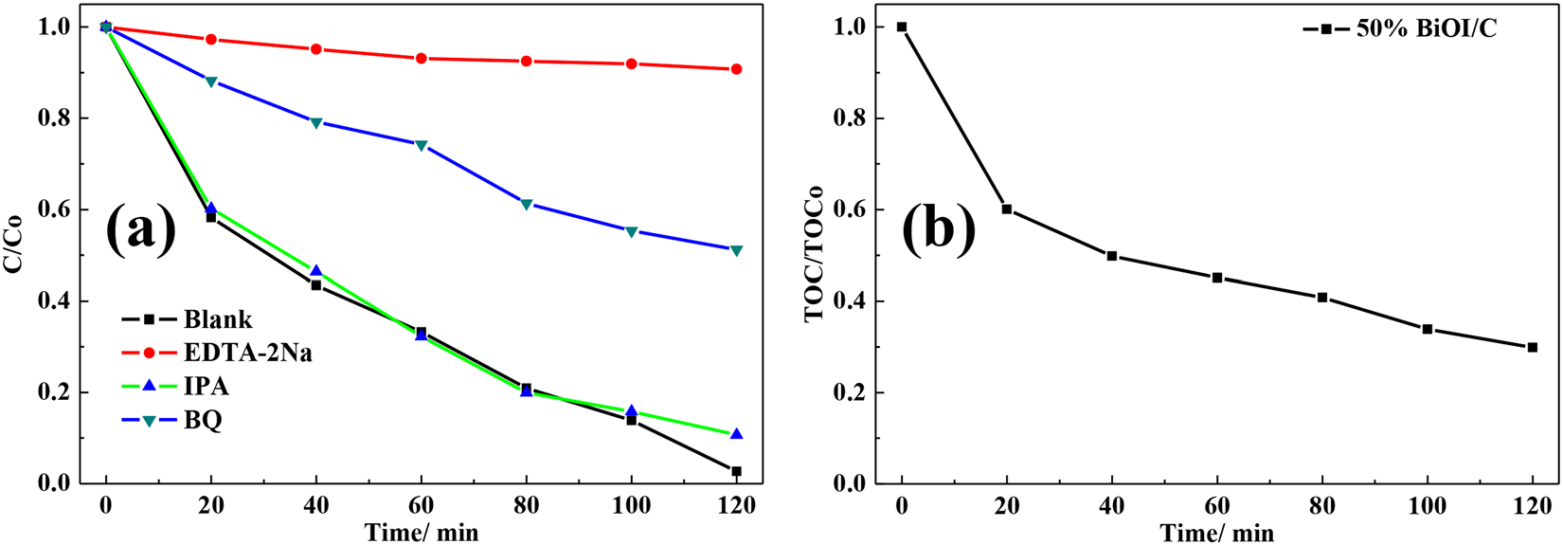
(**a**) Trapping experiments of active species and (**b**)TOC removal of RhB species of 50% BiOI/C during photocatalytic time.

To investigate the reaction mechanism during photocatalysis process, trapping experiment of active species (Fig. 8a) for 50% BiOI/C was evaluated by adding different agents, including isopropyl alcohol (IPA) for ^**·**^OH, p-benzoquinone (BQ) for O_2_^**·−**^ and edta-disodium (EDTA-2Na) for h^+^, respectively. After adding IPA as the trapping agent, for the photocatalytic activity, there was no obvious difference between control and IPA experiments. The results indicated the ^**·**^OH radical was not the main active species in photocatalysis process. However, when BQ and EDTA-2Na were selected as the trapping agents, the photocatalytic activity exhibited significantly decline, demonstrating the key roles of O_2_^**·**−^ and h^+^ in the degradation process. Moreover, the mineralization of 50% BiOI/C in degrading RhB was detected by TOC and showed in Fig. 8b. With expanding of illumination time, the removal ratio shows a gradually increase and reaches to about 71% in 120 min, meaning that most of the organic pollutant has been decomposed to mineral substances. At the same time, some of the pollutants exist in the form of intermediate products, so the TOC results show a slightly lower than the photocatalysis results. The valence band maximum (VBM) of BiOI (Figure S6) was calculated to be 1.36 eV by the correction value of 0.63 eV for normal hydrogen electrode (NHE) at pH 7. The band gap (E_g_) of BiOI was determined to be 1.78 eV according to plots of (αhv)^1/2^ versus h. Therefore, the conduction band minimum (CBM) was determined to be −0.42 eV by utilizing the formula E_CB_ = E_VB_ − E_g_, which is negative enough to generate O_2_^·−^ compared with the redox potentials of O_2_/O_2_^·−^ of −0.046 eV (*vs*. NHE)^5,22^. However, ^·^OH cannot be generated due to the negative VBM than the redox potentials of^·^OH/OH^−^, which was determined to be +2.38 eV^22^. The CB/VB level further demonstrates the result of trapping experiment.

**Figure 9:**
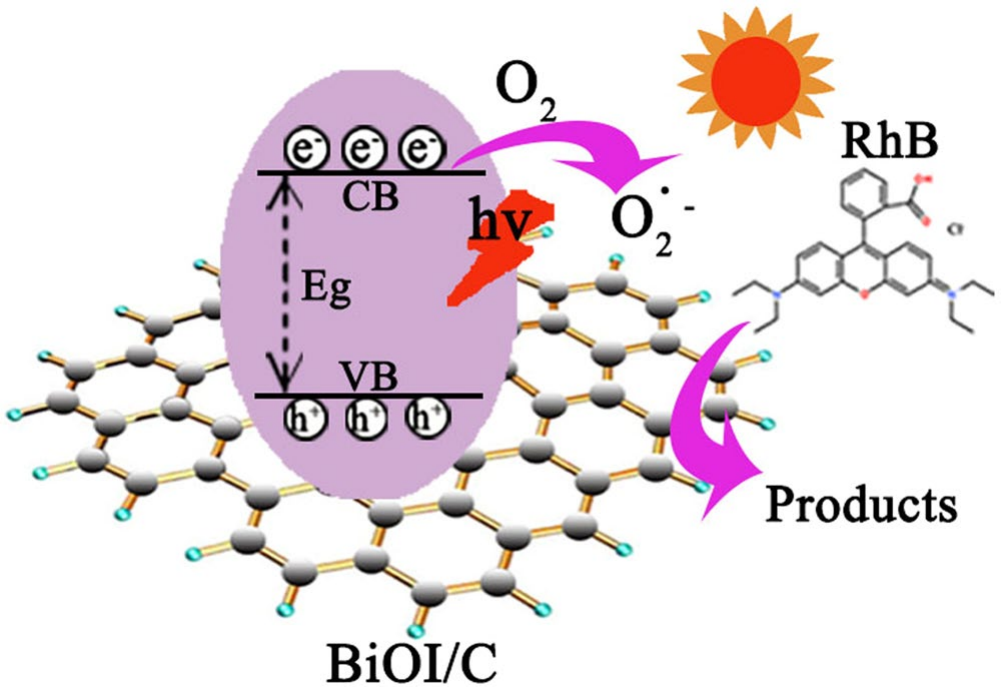
Mechanism schematic of BiOI/C in degrading RhB under visible light.

Thus, we proposed the reaction mechanism of photocatalysis process to review the crucial role of activated carbon (Fig. 9). Firstly, the YP-17D active carbon worked as absorption center and led to a higher visible light harvesting ability for photocatalysis. Secondly, the e^−^ on the valence band (VB) of BiOI was excited to the conduction band (CB), and the carbon could efficiently capture the e^**−**^ attributing to the bridge effect of Bi-C bonds, which not only inhibited the recombination of e^**−**^–h^**+**^ pairs, but also react with the absorbed O_2_ to generate O_2_^**·**−^. Thirdly, the optimized mesopores centered of 18.3 nm provide the pollutants with facile channel to arrive at the interface of BiOI/C, so the pollutants can be trapped within the effective functional mesoporous where photo-electrons accelerates the degradation process. Lastly, the hierarchical structures of BiOI/C endow them with high S_BET_ (145.6 m^2^/g) and adequate exposed reaction sites. The multiple synergistic effects above are responsible for the good photocatalytic activity.

## Conclusions

In this work, optimized micro-nano-hierarchical structure BiOI/C photocatalysts were successfully synthesized by a one-step green method. The red shift of absorption edge in BiOI/C may attribute to the growth of BiOI on the surface of carbon, which conduce to generate more O_2_^**·**−^ radicals for the photocatalysis process. The degradation rate of optimized BiOI/C toward RhB was 95% within 120 min, and the number is 3.36 times higher than that of pure BiOI. The reason for the high efficiency is that the enhanced photocatalytic activities deriving from the multiple synergistic effects of heterojunction, sufficient interfacial interaction, high S_BET_ (145.6 m^2^/g) and hierarchical structure. Especially, the hierarchical structure doping BiOI nanoparticles endowed the photocatalysts with easy separation of e^−^–h^+^ pairs, high quantum efficiency, adequate reaction sites and short diffusion pathways of pollutants, which is favorable for improving the photocatalytic activities. This work provides guidance in the facile fabrication of high performance BiOI/C based photocatalysts for degrading water pollutants.

## Methods

### Materials and Reagants

Bismuth nitrate [Bi(NO_3_)_3_·5H_2_O], ethylene glycol (EG) and potassium iodide (KI) were purchased from Sinopharm Chemical Reagent Co., Ltd. (Shanghai, China). The YP-17D activated carbon was bought from Shanghai Heatton Environmental Protection Technology Co., Ltd. All the chemicals were of reagent grade and were used without further purification.

### Sample Preparation

In a typical synthesis process of BiOI/C, two different solutions were prepared. Solution A: a certain amount of YP-17D activated carbon (1.5, 3.0 and 6.0 mmol) was dropped into a round bottom flask, followed by 3.0 mmol of Bi(NO_3_)_3_·5H_2_O and 60.0 mL of glycol. Solution B: 3.0 mmol of KI was dissolved into 20.0 mL glycol. After vigorous stirring at room temperature for 15 min, solution B was added to solution A and stirred to form a homogenous solution. The mixed solution continued to stir for 30 min and then transferred into 100 mL Teflon-lined stainless steel autoclaves for 12 hours at 120 °C.The obtained solution was centrifuged, washed with alcohol and distilled water and finally dried at 60 °C. The obtained materials were denoted as X-BiOI/C, and the value of X is the molar percentage of activated carbon (the wt% of carbon in 33%-BiOI/C, 50%-BiOI/C and 66%-BiOI/C were calculated as 1.7, 3.3 and 6.4%). The pure BiOI was synthesized following the reported method without adding carbon.

### Characterization

The crystal structures of theas-prepared materials were investigated by X-ray powder diffraction (XRD) (AXS D8 ADVANCE, Bruker) with Cu Kα radiation (λ = 1.5406 Å) and a scanning speed of 6°·min^−1^. The valence state of elements was tested by X-ray photoelectrons spectroscopy (XPS) (ESCALAB 250Xi, Thermo Fisher Scientific). The morphology of the samples was captured by the scanning electrons microscopy (SEM) (Zeiss_Supra55, Carl Zeiss AG) and high resolution transmission electrons microscope (HRTEM) (Tecnai G2 F30 S-Twin, FEI). The UV-visible diffuse reflectance spectroscopy (UV-Vis DRS) (TU-1901, PERSEE) was used to analyze the optical properties of samples. The specific surface area together with pore properties were calculated by the Brunauer-Emmett-Teller (BET) (ASAP 2460, Micromeritics) technique. The surface functional group’s information was measured by Fourier transforms infrared spectrometer (FT-IR) (TENSOR27, Bruker).

### Photocatalytic Evaluations

The photocatalytic activities of BiOI/C and pure BiOI were evaluated by the degradation of the RhB. The 500 W Xe lamp is used as a visible light source. Initial 10.0 mg of the as-prepared materials together with 50.0 mL of RhB aqueous solution (10.0 mg/L) were dropped into photocatalytic bottles. The mixed solutions were kept under stirring for 1 h to reach the absorption-desorption equilibrium in dark. Then the Xe lamp was turned-on after definite time (60 min), 3 mL of degraded solutions were taken out after every 10 min and centrifuged to remove the photocatalyst particles. The samples after centrifugation were analyzed by using UV-Vis spectrophotometer.

To investigate the active species generated during the photocatalysis process, the experiments of active species (^**·**^OH, O2^**·**−^ and h^+^) trapping were performed by adding 2 mmol/L isopropyl alcohol (IPA), 2 mmol/L benzoquinone (BQ) and 2 mmol/L ammonium oxalate (AO), respectively. Additionally, the TOC analysis was performed to detect the mineralization of organic molecules.

## Electronic supplementary material

Supplementary information

## References

[1] Shannon MA (2008). Science and technology for water purification in the coming decades. Nature.

[2] Fujishima K (1972). & Honda, Electrochemical photolysis of water at a semiconductor electrode. Nature.

[3] Zou ZG, Ye JH, Sayama K, Arakawa H (2001). Direct splitting of water under visible light irradiation with an oxide semiconductor photocatalyst. Nature.

[4] Luo B, Liu G, Wang LZ (2016). Recent advances in 2D materials for photocatalysis. Nanoscale.

[5] Di J (2016). Carbon quantum dots *in situ* coupling to bismuth oxyiodide via reactable ionic liquid with enhanced photocatalytic molecular oxygen activation performance. Carbon.

[6] Carey JH, Lawrence J, Tosine HM (1976). Photodechlorination of PCB’s in the presence of titanium dioxide in aqueous suspensions. Bull. Environ. Contam. Toxicol..

[7] Dahl M, Liu YD, Yin YD (2014). Composite titanium dioxide nanomaterials. Chem. Rev..

[8] Amano F, Yamakata A, Nogami K, Osawa M, Ohtani B (2008). Visible light responsive pristine metal oxide photocatalyst: enhancement of activity by crystallization under hydrothermal treatment. J. Am. Chem. Soc..

[9] Bi YP, Ouyang S, Umezawa N, Cao JY, Ye JH (2011). Facet effect of single-crystalline Ag_3_PO_4_ sub-microcrystals on photocatalytic properties. J. Am. Chem. Soc..

[10] Cheng HF, Huang BB, Dai Y (2014). Engineering BiOX (X = Cl, Br, I) Nanostructures for highly efficient photocatalytic applications. Nanoscale.

[11] Zhang YL (2014). Graphene-wrapped Bi_2_O_2_CO_3_ core-shell structures with enhanced quantum efficiency profit from an ultrafast electron transfer process. J. Mater. Chem. A.

[12] Xia JX (2015). Ionic liquid-induced strategy for carbon quantum dots/BiOX (X = Br, Cl) hybrid nanosheets with superior visible light-driven photocatalysis. Appl. Catal. B-Environ.

[13] Guan M (2013). Vacancy associates promoting solar-driven photocatalytic activity of ultrathin bismuth oxychloride nanosheets. J. Am. Chem. Soc..

[14] Bhachu DS (2016). Carmalt, Bismuth Oxyhalides: Synthesis, Structure and Photoelectrochemical Activity. Chem. Sci..

[15] Su J, Xiao Y, Ren M (2014). Direct Hydrolysis Synthesis of BiOI Flowerlike hierarchical structures and its photocatalytic activity under simulated sunlight irradiation. Catal. Commun..

[16] Ye KH (2015). BiOI-BiVO_4_ Photoanodes with significantly improved solar water splitting capability: p–n junction to expand solar adsorption range and facilitate charge carrier dynamics. Nano Energy.

[17] Li HP (2014). Enhanced visible light photocatalytic activity of bismuth oxybromide lamellas with decreasing lamella thicknesses. J. Mater. Chem. A.

[18] Di J (2014). Reactable ionic liquid-assisted rapid synthesis of BiOI hollow microspheres at room temperature with enhanced photocatalytic activity. J. Mater. Chem. A.

[19] Hahn NT, Hoang S, Self JL, Mullins CB (2012). Spray pyrolysis deposition and photoelectrochemical properties of n-type BiOI nanoplatelet thin films. ACS Nano.

[20] Li X, Yu JG, Jaroniec M (2016). Hierarchical photocatalysts. Chem. Soc. Rev..

[21] Zheng CR, Cao CB, Ali Z (2015). *In situ* formed Bi/BiOBr_x_I_1−x_ heterojunction of hierarchical microspheres for efficient vsible-light potocatalytic activity. Phys. Chem. Chem. Phys..

[22] Di J (2016). Bidirectional acceleration of carrier separation spatially via N-CQDs/atomically-thin BiOI nanosheets nanojunctions for manipulating active species in a photocatalytic process. J. Mater. Chem. A.

[23] Huang H (2015). Fabrication of multiple heterojunctions with tunable visible light-active photocatalytic reactivity in BiOBr−BiOI full-range composites based on microstructure modulation and band structures. ACS Appl. Mat. Interfaces.

[24] Liu H, Cao WR, Su Y, Chen Z (2013). Bismuth oxyiodide-graphene nanocomposites with high visible light photocatalytic activity. J. Colloid. Interf. Sci..

[25] Xu Z (2012). Decoration of BiOI quantum size nanoparticles with reduced graphene oxide in enhanced vsible-light-driven photocatalytic studies. Appl. Surf. Sci..

[26] Zhu LP (2016). *In situ* synthesis of N-doped carbon nanotubes–BiOCl nanocomposites and their synergistic photocatalytic performance. RSC Adv..

[27] Wang JC (2016). Indirect Z-Scheme BiOI/g-C_3_N_4_ photocatalysts with enhanced photoreduction CO_2_ activity under visible light irradiation. ACS Appl. Mater. Interfaces.

[28] Di J (2014). Preparation of sphere-like g-C_3_N_4_/BiOI photocatalysts via a reactable ionic liquid for visible-light-driven photocatalytic degradation of pollutants. J. Mater. Chem. A.

[29] Shu D (2014). BiOI-based photocactivated fuel cell using refractory organic compounds as substrates to generate electricity. Catal. Today.

[30] Hou JH, Cao CB, Idrees F, Ma XL (2015). Hierarchical porous nitrogen-doped carbon nanosheets derived from silk for utrahigh-cpacity bttery aodes and spercapacitors. ACS Nano.

[31] Hou JH (2014). From rice bran to high energy density supercapacitors: A new route to control porous structure of 3D carbon. Sci. Rep..

[32] Hou JH, Cao T, Idrees F, Cao CB (2016). A co-sol-emulsion-gel synthesis of yunable and uniform hollow carbon nanospheres with interconnected mesoporous shells. Nanoscale.

[33] Pan ML, Zhang HJ, Gao GD, Liu L, Chen W (2015). Facet-dependent catalytic activity of Nanosheet-Assembled Bismuth Oxyiodide Microspheres in Degradation of Bisphenol A. Environ. Sci. Technol..

[34] Di J (2014). Facile fabrication and enhanced visible light photocatalytic activity of few-layer MoS_2_ coupled BiOBr microspheres. Dalton Trans..

[35] Wang JL, Yu Y, Zhang LZ (2013). Highly efficient photocatalytic removal of sodium pentachlorophenate with Bi_3_O_4_Br under visible light. Appl. Catal., B.

[36] Chang C, Zhu L, Wang S, Chu X, Yue L (2014). Novel mesoporous graphite carbon nitride/BiOI heterojunction for enhancing photocatalytic performance under visible-light irradiation. ACS Appl. Mater. Inter.

[37] Wang YZ (2014). Electrostatic self-assembly of BiVO_4_-reduced graphene Oxide nanocomposites for highly efficient visible light photocatalytic activities. ACS Appl. Mater. Interfaces.

[38] Su J, Xiao Y, Ren M (2014). Direct Hydrolysis Synthesis of BiOI flowerlike hierarchical structures and its photocatalytic activity under simulated sunlight irradiation. Catal. Commun..

[39] He Z (2014). BiOCl/BiVO_4_ p–n Heterojunction with enhanced photocatalytic activity under visible-light irradiation. J. Phys. Chem. C.

[40] Huang YC (2014). Oxygen vacancy induced bismuth oxyiodide with remarkably increased visible-light absorption and superior photocatalytic performance. ACS Appl. Mater. Interfaces.

[41] Zhang B (2013). Rapid adsorption properties of flower-like BiOI nanoplates synthesized via a simple EG-assisted solvothermal process. J. Nanopart. Res..

[42] Xie XQ, Katja K, Wang GX (2015). Advances in graphene-based semiconductor photocatalysts for solar energy conversion: fundamentals and materials engineering. Nanoscale.

[43] Zhang N (2011). Assembly of CdS nanoparticles on the two-dimensional graphene scaffold as visible-light-driven photocatalyst for selective organic transformation under ambient conditions. J. Phys. Chem. C.

[44] Hou YD (2013). Chorkendorff, layered nanojunctions for hydrogen-evolution catalysis. Angew. Chem., Int. Ed..

[45] Lei YQ (2010). Room Temperature, Template-free synthesis of BiOI hierarchical structures: visble-light photocatalytic and electrochemical hydrogen storage properties. Dalton Trans..

[46] Huang HW, Liu K, Chen K, Zhang YH, Tian N (2014). Tunable 3D hierarchical graphene-BiOI nanoarchitectures: their *in situ* preparation, and highly improved photocatalytic performance and photoelectrochemical properties under visible light irradiation. RSC Adv..

[47] Huang HW, He Y, Du X, Chu PK, Zhang YH (2015). A General and facile approach to heterostructured core/shell BiVO_4_/BiOI p-n junction: room-temperature *in situ* assembly and highly boosted visible-light photocatalysis. ACS Sustain. Chem. Eng..

[48] Di J (2016). Carbon quantum dots induced ultrasmall BiOI nanosheets with assembled hollow structures for broad spectrum photocatalytic activity and mechanism insight. Langmuir.

