# Supplementary material for: Micro and nano hierachical structures of BiOI/activated carbon for efficient visible-light-photocatalytic reactions
Source: Sci Rep. 2017 Sep 15;7:11665. doi: 10.1038/s41598-017-12266-x (PMC5601909; doi:10.1038/s41598-017-12266-x)
Supplement: Supplementary file 1 — Supplementary information [file 41598_2017_12266_MOESM1_ESM.doc]

**Supporting Information**

Micro and nano hierachical structures of BiOI/activated carbon for efficient visible-light-photocatalytic reactions

Jianhua Hou^1,2^, Kun Jiang^2^, Ming Shen^2*^, Rui Wei^1^, Xiaoge Wu^1^, Faryal Idrees^3^ and Chuanbao Cao^3^

^1^ *Jiangsu Key Laboratory of Environmental Material and Engineering,* *College of Environmental Science and Engineering, Yangzhou University, Yangzhou, 225127, P. R. China*

^2^ *College of Chemistry and Chemical Engineering, Yangzhou University, Yangzhou, 225002, P. R. China*

^3^ *Research Centre of Materials Science, Beijing Institute of Technology, Beijing 100081, P.R. China*

Correspondence and requests for materials should be addressed to M. S. (**shenming@yzu.edu.cn**)


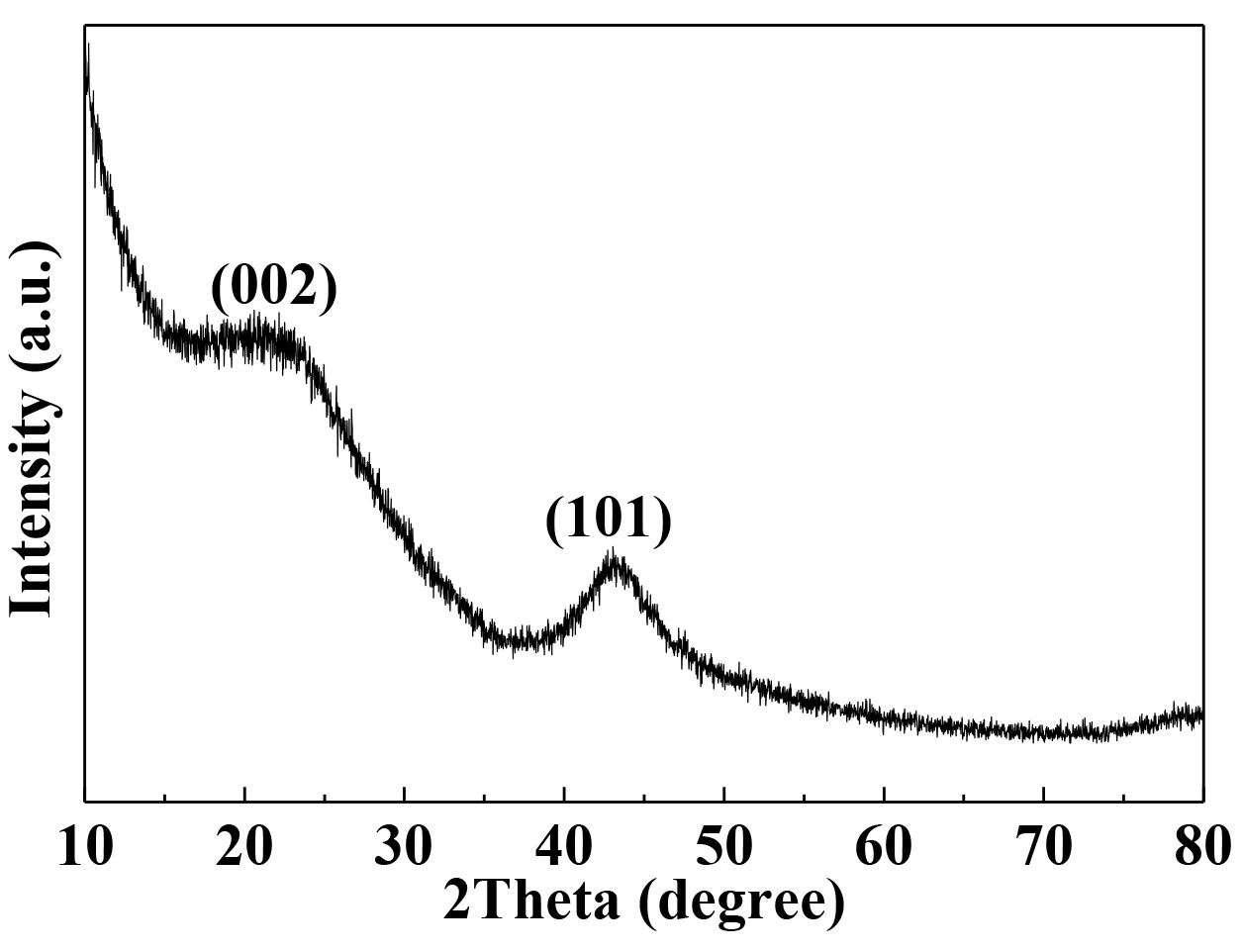


**Figure S1.** XRD pattern of YP-17D activated carbon.

The patterns of activated carbon exhibit two broad peaks at 22° for (002) and 43° for (101) plane, which correspond to the previous study, indicate that they are the amorphous carbon ^1^.


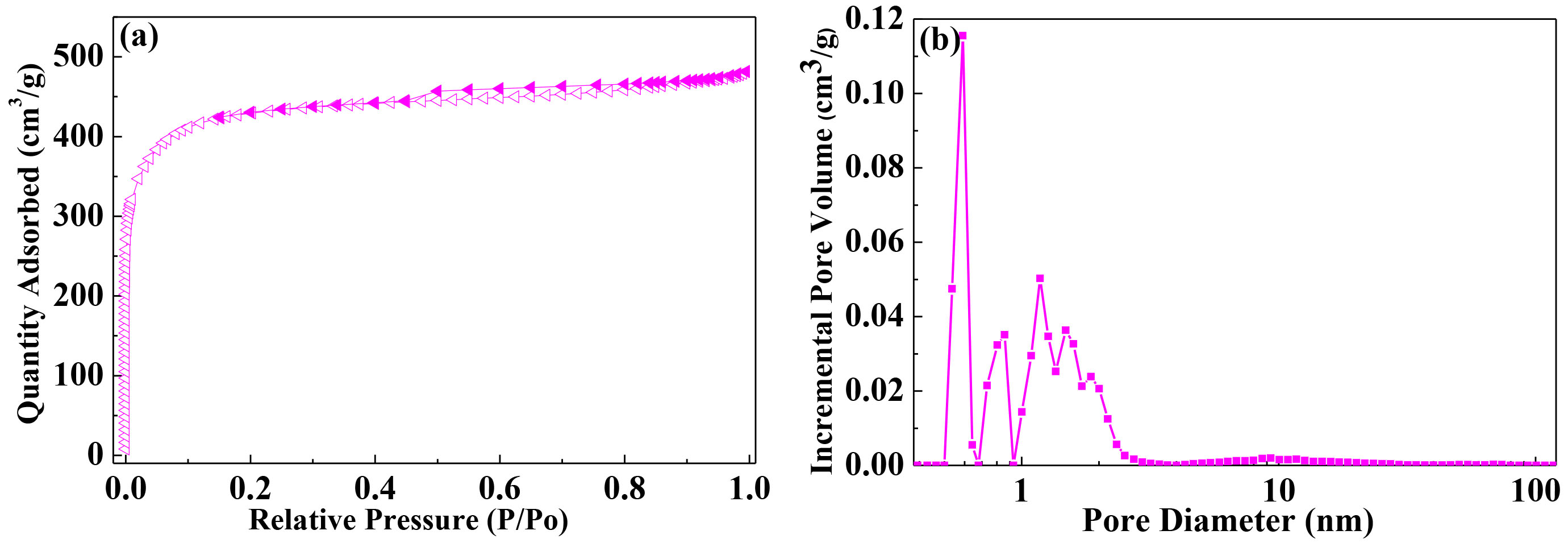


**Figure S2.** (a) Nitrogen adsorption-desorption isotherm (b) pore size distribution of YP-17D activated carbon.

The nitrogen adsorption-desorption isotherm of YP-17D (Figure S2a) is consistent with type I curve^2-4^, indicating the typical micropores feature. The pore size distribution curve (Figure S2b) shows that pore of YP-17D center at 0.61 and 0.85 nm and continue distribute within the range of 1~3 nm. The S_BET_ is 1660 m^2^/g and the pore volume is 0.74 cm^3^/g.

**
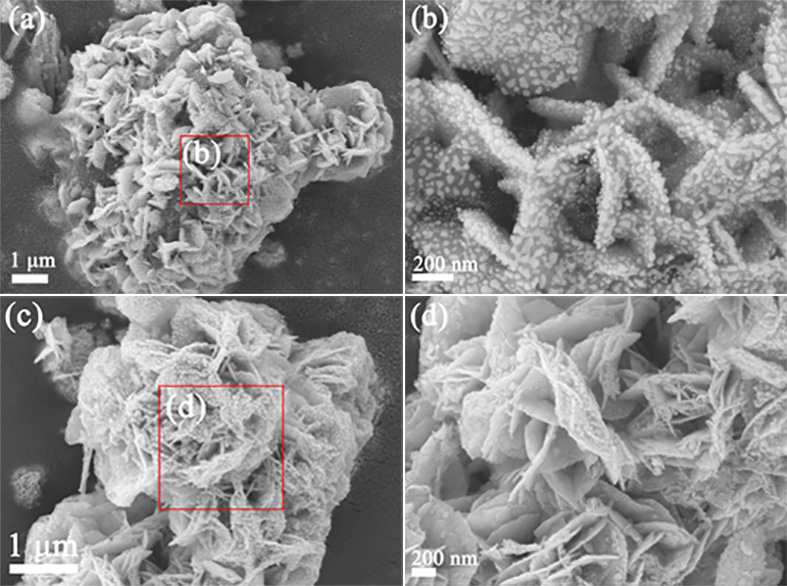
**

**Figure S3.** SEM images of (a, b) 33%-BiOI/C and (c, d) 66%-BiOI/C

**
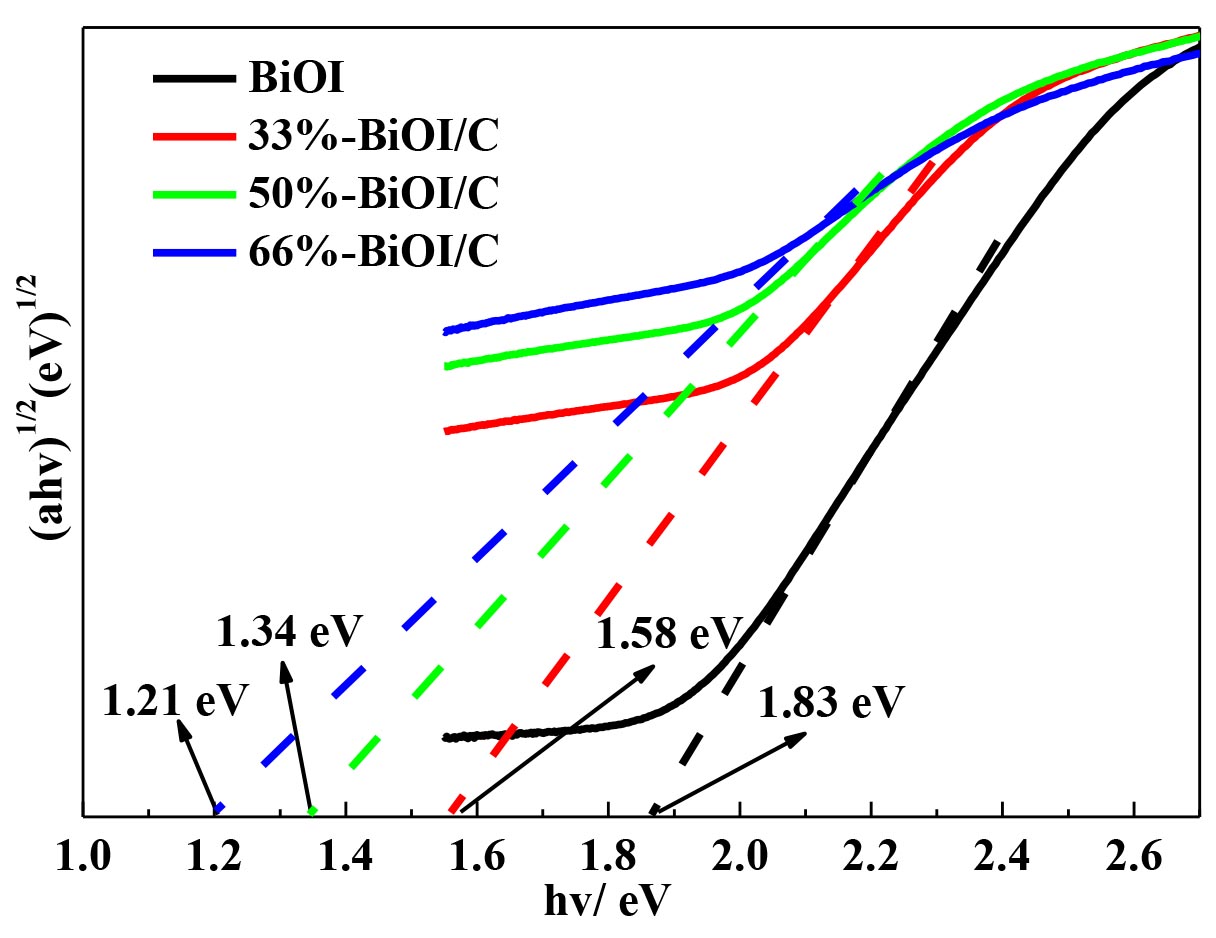
**

**Figure S4.** The plots of (αhv)^1/2^ versus hv of BiOI and X-BiOI/C

**
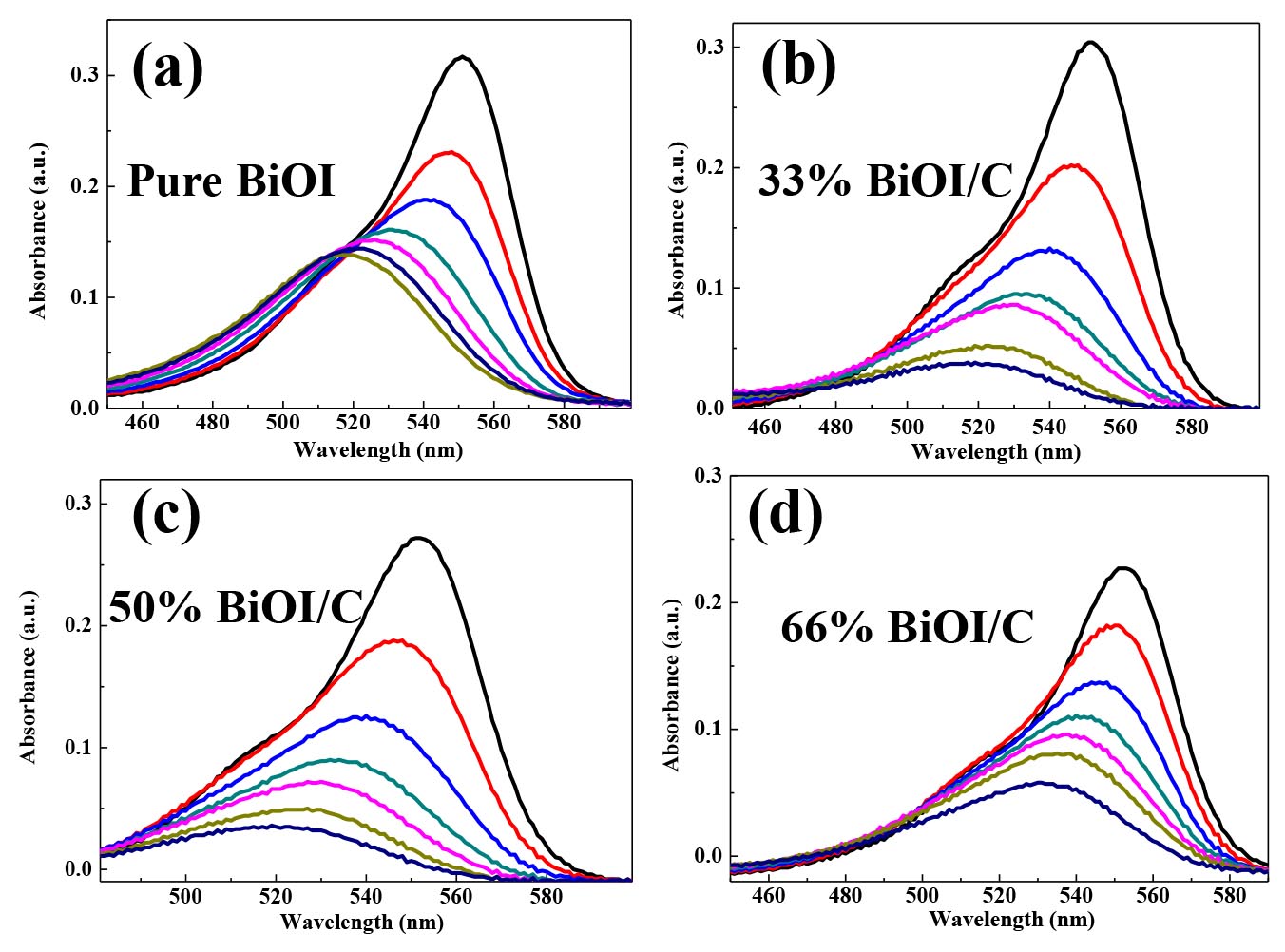
**

**Figure S5.** The plots of (αhv)^1/2^ versus hv of BiOI and X-BiOI/C


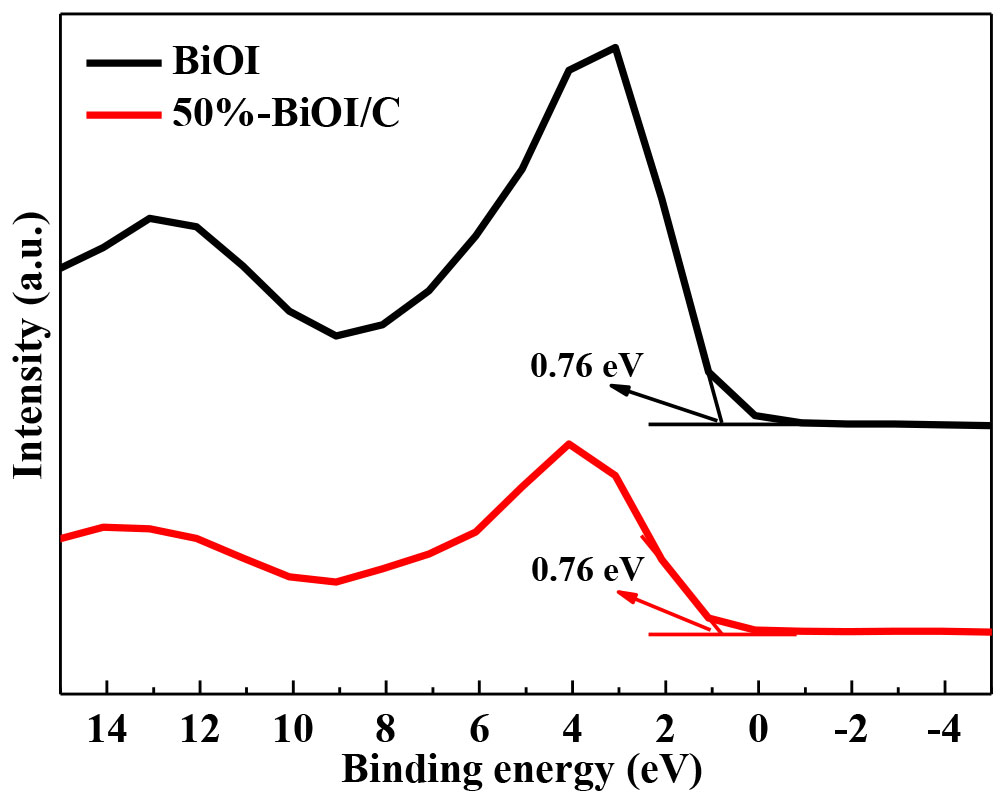


**Figure S6.** Valence-band XPS spectra of BiIO and 50%-BiOI/C

Table S1. Percentage of pore volume in BiOI and BiOI/C.

| Pore size | BiOI | 33% BiOI/C | 50% BiOI/C | 66% BiOI/C |
| --- | --- | --- | --- | --- |
| Micropores(%) | 2.2 | 7.2 | 9.8 | 17.9 |
| Mesopores(%) | 83.6 | 74.8 | 65.3 | 57.8 |
| Macropores(%) | 14.2 | 18.0 | 24.9 | 24.3 |

Table S2. Percentage of pore area in BiOI and BiOI/C.

| Pore size | BiOI | 33% BiOI/C | 50% BiOI/C | 66% BiOI/C |
| --- | --- | --- | --- | --- |
| Micropores(%) | 14.0 | 28.7 | 33.4 | 36.2 |
| Mesopores(%) | 53.1 | 32.3 | 24.7 | 15.5 |
| Macropores(%) | 32.9 | 39.0 | 41.9 | 48.3 |

**References**

1. Hou, J. H., Cao, T., Idrees, F. & Cao, C. B. A co-sol-emulsion-gel synthesis of yunable and uniform hollow carbon nanospheres with interconnected mesoporous shells, *Nanoscale* **8**, 451-457 (2016).

2. Di, J., Xia, J. X., Ji, M. X., Xu, L., Yin, S., Chen, Z. G. & Li, H. M. Bidirectional acceleration of carrier separation spatially via N-CQDs/atomically-thin BiOI nanosheets nanojunctions for manipulating active species in a photocatalytic process, *J. Mater. Chem. A* **4**, 5051-5062 (2016).

3. Li, H. Q., Cui, Y. M. & Hong, W. S. High photocatalytic performance of BiOI/Bi_2_WO_6_ toward toluene and Reactive Brilliant Red, *Appl. Surf. Sci.* **264**, 581-588 (2013).

4. Akhavan, O. & Ghaderi, E. Photocatalytic Reduction of Graphene Oxide Nanosheets on TiO_2_ Thin Film for Photoinactivation of Bacteria in Solar Light Irradiation, *J. Phys. Chem. C.* **113**, 20214-20220 (2009).
